# Developing an Intervention to Improve the Health Related Quality of Life in Children and Young People With Serious Parental Mental Illness

**DOI:** 10.3389/fpsyt.2019.00155

**Published:** 2019-04-09

**Authors:** Judith Gellatly, Penny Bee, Adekeye Kolade, Diane Hunter, Lina Gega, Craig Callender, Holly Hope, Kathryn M. Abel

**Affiliations:** ^1^Faculty of Biology, Medicine and Health, Centre for Women's Mental Health, Manchester Academic Health Science Centre, The University of Manchester, Manchester, United Kingdom; ^2^Division of Nursing, Faculty of Biology, Medicine and Health, Manchester Academic Health Science Centre, School of Health Sciences, The University of Manchester, Manchester, United Kingdom; ^3^NSPCC, London, United Kingdom; ^4^Department of Health Sciences, Hull York Medical School, University of York, York, United Kingdom; ^5^St. Nicholas Hospital, Northumberland Tyne and Wear NHS Foundation Trust, Newcastle upon Tyne, United Kingdom; ^6^Greater Manchester Mental Health NHS Foundation Trust, Manchester, United Kingdom

**Keywords:** co-production, children, young people, serious mental illness, parents, intervention, health-related quality of life

## Abstract

**Introduction:** Children and adolescents living with parental mental illness (CAPRI) are at increased risk of behavioral, social and educational difficulties, mental and physical health problems and have poorer quality of life (QoL). Adverse outcomes can extend into adulthood but are not inevitable. Recent policy and stakeholder consultation recognize the urgent need for interventions that extend beyond objective, service-led measures of health. Systematic evidence synthesis has demonstrated a lack of evidence-based interventions for enhancing holistic, child-centered outcomes. We aimed to co-develop a manualised, community-based intervention to improve QoL in CAPRI. Precedence was given to the QoL domains that were prioritized by stakeholders and deemed feasible to modify within a health and social care context. We describe here the modeling phase of developing the intervention emphasizing co-production activities with CAPRI, their families and professionals who support them.

**Methods:** Semi-structured interviews and focus groups with CAPRI (*n* = 14), parents (*n* = 7), and professionals from health, social and educational sectors (*n* = 31) in the UK. Topic guides qualitatively explored participants prior experiences, unmet needs, perceived barriers and facilitators to receiving/delivering support, and their ideals for a new intervention. Findings were synthesized with existing research evidence and presented to a mixed panel of clinical academics and health and social care professionals. A consensus exercise was used to identify the preferred structure, format and content of the manualised intervention.

**Results:** An 8-week group intervention for 6–16 year olds and their parents, called Young SMILES, has been co-developed along with associated training materials for facilitators. Each session addresses an identified need, but is underpinned by cross-cutting themes pertaining to mental health literacy, parent-child communication, and problem solving skills. Sessions are delivered by two trained facilitators and held in accessible and acceptable community locations weekly for 2 h.

**Conclusion:** Young SMILES captures a broad age range and level of need for CAPRI and can be evaluated with quantifiable child-centered outcomes. In line with current policy directives, this is the first UK-based, multi-context intervention to improve QoL in this population. Implementation and referral mechanisms are currently being evaluated in a multi-site feasibility trial.

## Introduction

Children and adolescents living with parental mental illness (CAPRI) are poorly provided for in current social care and educational settings (1, 2). Children and young people (CYP) growing up in families affected by parental mental health disorders have an elevated risk of physical health (3–5) and mental ill health (6, 7), attention or educational difficulties (1, 8, 9) and emotional and behavioral problems (10–12). They may be more socially isolated (13), and some will experience neglect (14), family separation (15), or social care involvement (16). Longer term outcomes for these children extend into adulthood and include chronic psychological difficulties, social and occupational dysfunction and substance misuse (17).

Risk of adverse outcomes is of increasing public health concern. Population estimates from the United States, Sweden and Australia suggest that between 38 and 50% of women with serious mental illness (SMI) will be mothers and approximately 25% of men with SMI will be fathers (18). In the UK (and Sweden) 1 in 4 children at any time are living with parental mental illness and, by 16 years of age, with over 50% of children will have experienced parental mental disorder of a severity sufficient to present to services.

Adverse outcomes are not inevitable and the impact of parental mental illness on children's outcomes is modifiable. Data suggests that at least half of all children with a parent with mental illness may not experience any psychiatric symptoms (19), and only a small proportion will access mental health services (10). Individual and family resilience can be heavily determined by an ability to find positive meaning in challenging events, to recognize a need to change social interactions or environmental conditions, and the increased availability of health-sustaining resources. Thus, how a parent or child makes sense of their experiences of mental illness may be as, if not more, important than the actual experience itself.

There is a growing literature exploring community-based interventions for CAPRI which has been comprehensively reviewed (20). Traditionally, parents have been considered the primary change agent for their children and parent-centered interventions have been evaluated for secondary effects on child outcomes. Quantified outcomes have primarily been behavioral or psychological in origin, and mediated by improved parenting or enhanced parental health (11, 21). Meta-analysis suggests that such parent-centered interventions are of variable quality and reliability (20).

Increasingly child and family-based approaches are permeating research arenas and these interventions offer an alternative and promising avenue for change. Child-centered interventions seek to establish the child as the major change agent. Research and consultation has suggested that children often have a different view of their situation, and a different idea of what would help compared to parents or mental health workers (19, 22). A philosophical shift away from “children as patients” has led to clinical, therapeutic interventions being replaced by more strength-based approaches. Peer support interventions have been advocated as a possible means by which to provide respite and reduce the isolation for CAPRI but are often not standardized or time bounded to a degree that facilities their roll-out or implementation across resource-constrained services. Additional work is needed to agree intervention priorities, identify the most likely active ingredients, and deliver these in the most cost and time efficient format.

In evaluating adult and services, focus has moved from simply the absence of disease to a more holistic approach, recognizing that improving the QoL, and specifically health-related QoL (HRQoL) as has great significance (23). QoL concerns how an individual perceives their own well-being and life experiences with respect to their personal beliefs, goals and expectations (24). In the context of severe parental mental illness, it can thus be conceptualized as both outcome and a modifier of children's short- and longer-term resiliency.

Multi-dimensional models of QoL reflect the scope and complexity of an individual's QoL judgments and offer a coherent, empirical based framework through which to potentially maximize intervention impact by simultaneously addressing multiple QoL domains. Targeted approaches to modifiable dimensions of QoL have the potential to improve the shorter and longer term availability of internal assets as well as external resources. These combined resources can strengthen protective factors, counteract or moderate risk factors and help to achieve positive adaptation in the face of adverse life experience (25–30). Effective adaptation can in turn, precipitate improved QoL appraisals, creating a positive feedback loop and maximizing return on investment.

Systematic evidence synthesis has demonstrated a striking lack of evidence-based interventions for enhancing holistic, child-centered outcomes. Bee et al. (20) identified only three trials focused on CYP with severe parental mental illness; and none of these explored QoL outcomes of CAPRI. This knowledge provides a compelling argument for a focused exploration of CAPRI need and the theoretical development, delivery and evaluation of novel, effective interventions (2, 20, 31). This paper describes development of a new intervention called Young SMILES [Simplifying Mental Illness and Life Enhancing Skills; (32)]. Development of the intervention was conducted in line with the Medical Research Council (MRC) complex interventions framework. Following identification of existing evidence and theory development, the MRC framework stipulates modeling an intervention by identifying key components of the intervention and its evaluation prior to conducting a feasibility trial (33).

## Materials and Methods

Co-development of the intervention involved three phases: Phase 1—Needs analysis—review of existing literature and primary qualitative research with stakeholders to identify needs and preferred delivery models; Phase 2—stakeholder consensus—professional synthesis exercise to identify emerging themes and agree provisional content and delivery preferences and; Phase 3—manual development and refinement—locating stakeholder preferences in existing knowledge literature (20) and theory of change involving team work.

A planning group of research team members, including those with clinical and service delivery experience, met face-to-face monthly throughout the project to discuss existing intervention literature and materials, emerging research data and the development of the Young SMILES intervention. Additional remote meetings were held as required for manual development.

The activities and findings from each development activity follow.

### Phase 1 Needs Analysis

#### Existing Literature

The work presented here reports on a commissioned piece of work that aims to shift the spotlight away from the medicalization of CAPRI to focus on the development of a child-centered, community-based intervention to improve quality of life for all children affected by severe parental mental illness.

Literature exploring community-based interventions for CAPRI has been recently and comprehensively reviewed (20). This review, and the intervention resources identified with it, was used as a starting point for intervention development. An existing intervention, The Family SMILES intervention (34), provided a starting template for intervention development. Family SMILES is based on the SMILES programme, a 3-day intervention for Australian CYP aged 8–16 with a parent experiencing mental ill health (35). SMILES has been evaluated positively with respect to improving CYPs knowledge of mental health and coping skills (36).

Family SMILES has been piloted in the UK but not yet rigourously evaluated. FAMILY SMILES takes a deliberately narrow approach; focusing only on CYP at-risk of maltreatment or neglect. The intervention comprised 8 weekly groups: 6–8 CYP sessions; 6 one-to-one weekly sessions with parents; and a final CYP-parent joint session with each family. Its aim was to enhance children's resilience and self-esteem and parents' protective function; and to improve parent-child communication and family relationships. Preliminary evaluation of Family SMILES has highlighted potential benefits for CAPRI in increased social functioning and confidence, reduced social isolation and reduced blame associated with parental illness. For parents, benefits included less distress and unhappiness, a shift of thinking from own needs to those of their children; and for families overall a more relaxed atmosphere, openness about parental mental illness, empathy between CAPRI and parents and shared responsibilities (34).

#### Agreeing the Working Aim

The working aim, agreed between the research team and the project steering group, was to broaden the scope and content of Family SMILES to make it specific to families whose parents have SMI; to make it applicable to a wider age-range of CAPRI, to align it with NHS priorities and service structures and to make it deliverable in different practice settings by a varied staff skill mix, including NHS and voluntary sector providers. In the context of the UK NHS, our intention was to co-create a child-centered approach with far broader reach (geographical and across ages and needs), with a specific focus on enhancing children's QoL. QoL was defined according to a published, empirically-led model derived specifically for the target population (37). This definition upheld QoL as a multi-dimensional construct comprising of 5 domains spanning emotional, physical and social well-being, family context and experience and children's self-esteem and self-actualization. Precedence was given to three domains identified as priority by stakeholders and potentially capable of being modified via a time bounded health service intervention. These domains comprised emotional and social wellbeing, family experience and self-esteem and actualization. A fourth domain, physical wellbeing was represented as a secondary goal of the intervention and targeted through in-session education.

Within these priorities the importance of improving problem-based coping skills, increasing mental health literacy, and alleviation of parental mental health symptoms were evident.

Recognizing the importance of stakeholders' voices, our explicit intention was to place childrens' needs at the center of the process to create a best evidence, feasible and acceptable intervention to CAPRI improve health-related QoL (2). We used this approach to ensure that a priori beliefs and existing Family SMILES components did not drive our assumptions about what would and would not work for children. To achieve this, we interviewed participants blind to the Family SMILES intervention and explored ways to support CAPRI directly and separately from the experiences and needs of their parents. Despite the incidence rates of mental illness being increased for CAPRI, as a large percentage will not experience mental illness, we anticipated an intervention relevant to all CAPRI.

#### Primary Research

Stakeholders, including children and adolescents, parents and practitioners from NHS and voluntary settings (including managers), were invited to participate in discussion groups and individual interviews if they preferred. Individual discussion groups were held for each stakeholder group to maximize opportunities for participation. Consent was taken from all participants prior to the discussion group/interview commencing. Parents were asked to consent for their child or adolescent taking part. For CYP, parental/guardian consent was required in addition to their assent. All parents/guardians approached agreed for their child to take part. Participation of both CYP and their parent was not necessary, but all were invited. Practitioners in the recruiting sites initiated contact with families and assisted in obtaining consent. Participants were asked to complete a short demographic questionnaire at the beginning of the discussion (separate questionnaires were developed for each stakeholder group). Discussion groups and individual interviews were held at a community location convenient to the participants. Travel expenses were reimbursed and refreshments provided.

Attendees were informed about the aim of the study and the value of their involvement in developing a new intervention to improve the QoL of CAPRI. The terminology used was altered for each stakeholder group to ensure understanding. Semi-structured topic guides explored experiences of previous support, unmet needs, barriers and facilitators to receiving/delivering support, gaps in current care and what an ideal intervention would look like. Interviews and focus groups were audio recorded or notes were taken. For CYP, methods to enhance engagement, such as using post-it notes to provide views anonymously for discussion, emojiis to express feelings about aspects of their ideal intervention and pens to draw were implemented. Data were analyzed using thematic analysis (38).

Interviews and focus groups took place between June and October 2016. Participants were recruited via two different voluntary organization regional branches (A and B) and the NHS (practitioners only). Some had direct experience of Family SMILES. Table 1 provides an overview of the data collection methods and participant demographics.

**Table 1:** Stakeholder consultation methods and participants.

	**Young People (*n* = 14)**	**Parents (*n* = 7)**	**Practitioners (*n* = 31)**
Method:			3
Focus Group (number conducted)	2	1	
Interview (number conducted)	2 face-to-face	2 telephone	
			0
Family SMILES experience (n, %)	Previous experience 6, 43% No previous experience 8, 57%	No previous experience 7, 100%	Previous experience: Voluntary organization A practitioners (10, 32%) No previous experience: Voluntary organization B practitioners (15) and; NHS practitioners (6) (68%)
Gender	9 girls (64%), 5 boys (36%)	7 women (100%)	28 women, 1 man
Age (mean, range)	11, 10–16	41.14, 33–47	
Siblings (*n*, %)	11, 79%		
Live with parent experiencing SMI (*n*, %)	13, 93%		
Awareness of parental mental health type (n, %)	10, 71%		
Number of children under 17 years (mean)		2.43	
Time experienced SMI (mean, range)		14, 2–25	
Professional qualifications			Social work, teaching, counseling, clinical psychology, OT, mental health nursing, family therapy
Numbers in managerial position (*n*, %)			7, 23%

Key themes were identified in relation to the purpose and composition of the Young SMILES intervention with comparisons between participant groups recognized. Core content topics or themes identified across all stakeholder groups highlighted that Young SMILES should educate (improve mental health literacy); reduce isolation and support. A theme about delivery preferences was also documented.

The following provides a summary of these findings and evidence of differing viewpoints.

#### Educative—Improving MH Literacy

Improving mental health literacy was regarded as important by young people and parents; and considered a key element of Young SMILES. This related to one's own understanding but also understanding within the wider society. For parents, lack of understanding of their own problems was acknowledged as having an impact on their views and beliefs about their ability successfully to undertake their role within the family. One parent highlighted the effect that depression can have and how improving their understanding is needed for help seeking.

“*I understand what depression is now I can see it coming and see it in other people but the very first time you're not sure if it's … you question yourself a lot. You question whether you're a good mum. Is it really you that's annoying everyone and people pick up on the signs. So at the beginning of the depression it's really understanding that you have got a mental illness yourself and recognizing it and then going to the doctors to do something about it without feeling embarrassed or stupid. That's a really big part of mental health.”* (Parent)

There was a general sense, particularly among CYP, that society lacked awareness of what the experience of having a parent with an SMI is like. The negative consequences of this had an effect on their daily lives.

“*no-one knows what I'm dealing with, they bully me because of my mum, they know I'm a young carer.”* (CYP)

Attending school was challenging for many; lack of understanding and awareness of the effects of having a parent with an SMI extended beyond their peer group to their teachers. CYP were eager for changes to occur:

“*I want them [school] to understand more, what I am as a young carer going through…understanding of why I come in late.”* (CYP)

Whilst it was acknowledged that the intervention could not necessarily change society's awareness and perceptions, professional stakeholders recognized the importance of educating children and teachers about their parent's mental illness and saw this as a key element of providing support and overcoming the challenges those children can face:

“*And that's often leaving children with a lot of space to create their own ideas of what's going on which is often much more frightening in the reality isn't it?”* (NHS practitioner)

“*it was quite a normal reaction for the children to be able to want to understand their mother's difficulties which had been quite pronounced and defined really.”* (NHS practitioner).

#### Reducing Isolation

As a result of living with a parent experiencing SMI, children reported that they often felt emotionally isolated. These experiences extended to their social lives, where some sacrificed activities for friends to look after their parent(s).

“*I just can't go out with friends – need to make sure mum is OK first.”* (CYP)

Parents acknowledged this was often the experience of their children and that there was often a reversal of roles, with their children taking on responsibilities they felt they shouldn't. This was distressing for parents, who felt a sense of guilt because of their inability to parent:

“*you're having this problem you cannot get yourself out of, when you look at your children, you cannot help them.”* (Parent)

“*mental health becomes who you are.”* (Parent)

Consistent with these experiences, professionals acknowledged the difficulties that CAPRI experience emotionally, highlighting their inability to understand the root cause.

“*Yeah, they don't understand their own emotions, they don't understand the parents' emotions. They think that their parents' emotions are a reaction to their behavior, which sometimes that is what's going on, and the child takes all the responsibility for that.”* (Voluntary organization A practitioner)

It was acknowledged that an intervention focused on children's needs has to negotiate the sensitivity of helping parents understand that their illness and its behaviors may adversely influence their children.

“*It's that introductory process really, around, not just introducing what [the intervention] is about, but you're introducing the concept that there's some idea that this issue, that this parent has probably been living with for 20 years or more, can potentially impact on their children, and impact on how they are parenting their children.”* (Voluntary organization A practitioner)

#### Support

Practitioners recognized the need to provide support to CYP and parents. For all stakeholders, the value of support on a predominantly emotional, but at times practical level was considered important. The value of providing a group intervention including peers was recognized as a key element. Many saw it as a way of reducing isolation among CYP and parents. Reflecting upon feedback from families they had worked with previously, one practitioner stated:

“*It's ultimately about I thought it was only us who was struggling. I thought it was only me that at times hates my mum. And from a parent's perspective, I thought it was only me that is really struggling with my teenager, and all those issues. So I think there's also that experience really, about coming together and that mutual support and that I'm not on my own. And that there are similar experiences shared really.”* (Voluntary organization B practitioner)

The additional value that involving their parent in an element of the intervention would provide was thought of positively across both sectors, with additional benefits for the family identified:

“*And I think permission as well, I think permission to have conversations, that families…that they might be thinking of in their heads but not actually have the courage or feel they've got the permissions to have those conversations with each other.”* (Voluntary organization B practitioner)

The importance of helping parents to overcome their unwillingness to approach difficulties via a more family or child-centered approach was acknowledged. Practitioners identified that they could play a supportive role to promote parent engagement:

“*…my experience is often parents don't want you to, or they're maybe a little bit more reluctant, to have children involved say with the family meeting. And children will often be at school when people are calling…I sense there would be a reluctance and so some of the skill is about connecting to parents I think initially and maybe doing some work there about what maybe could be talked about and things and whether they can do some of that and we can support them in doing that.”* (NHS Practitioner)

Parents recognized that talking to their children about mental health was challenging; particularly when they were “*really bad*” and that providing their child with the opportunity to speak to other CYP in similar circumstances helped their child to address some of the issues they were facing via different supportive avenues.

“*It's like when you're talking to your own friends isn't it, you can open to your best friends and your partner, and whatever. But sometimes it's like, well, I can tell her anything but not about the mental health because you don't want them panicking…But when they're together and they've been through the same experiences…they can open up and they can say, my mum's done that. And then your [other parent's child] little one might say, well, my mum does that as well, or, you know, stuff like that, so they're not alone.”* (Parent)

CYP and parents valued having the opportunity to have some “respite.” Within the discussions it was evident that there were similarities between the views of parents and CYP such as the recognition of the importance of retaining their family unit but subtle differences were identified. CYP expressed a desire to receive support in an environment separate to their parents, for the most part, to discuss the difficulties they were experiencing and they felt being away from parents would make it easier for them to do so and reduce any impact this may have upon their parent:

“*Or like anything's happening, any bullying or anything like that, if it did happen to me, I'd rather speak to [voluntary organization] about it than my mum because I don't want to put that pressure on my mum and everything…if it's something that's gone on at school or something that's happened, I have to keep it to myself. If I did keep it to myself, I would be okay with it, but then I'd just get a bit worried.”* (CYP)

“*My mum makes me nervous”. (CYP)*

Parents mirrored these views, identifying the importance of ‘*children having a separate opportunity*’ (Parent), but also recognized that, at times, their children's sense of responsibility for them was overwhelming and became a barrier to engaging in activities without them:

“*I think it would be better if the children were separate and they had, like, a little group together and then all together. I know when our [child's name] had a first referral to [the voluntary organization] and she [facilitator] was, like, come with me. I said, she's dead nice. Because I'd spoken to her before and we'd gone through it all. I said, she's dead nice, she's lovely. And our [child's name] usually…if you meet her, she's, like, hello. And she was, like, no mum. And she got hold of my hand, you're coming with me. And I'm, like, are you sure you want me with you? … We're both turning up, she doesn't want to leave me by myself*.” (Parent)

### Delivery Preferences

For CYP, it was important that they had “*fun*” and that despite Young SMILES being an opportunity to learn more about their parents' mental health, it was vital that did not mirror the school environment, offering an opportunity to learn in non-traditional formats.

“*Instead of just sitting down and talking…doing an activity…engaging in a different way so it doesn't feel like you are in school.” (CYP)*

CYP focused their discussions on activities that could be included to ensure it was “fun.” They wanted an opportunity for relevant team building and physical activities and somewhere to share their own experiences. Older children additionally expressed the value of “*anonymous self-expression*.” Whilst they recognized the potential use of technology in the activities included in the Young SMILES sessions, they were reluctant for it to be incorporated because of its potential effect on their ability to engage with others in the group highlighting it was “*anti-social” and reflecting on the impact that involving technology may have upon the intervention:*

“*It's [technology] wasting our time together.” (CYP)*

While parents acknowledged the need for “*fun*” and allowing their children to experience “*childhood*” they focused less on the type of activities they thought Young SMILES should contain; predominantly exploring the potential outcomes that could be achieved for their children such as encouraging independence/increase confidence, educating and normalizing:

“*Because they kind of have that attachment to us [our children] and that worry, that anxiety. And that's true really, because they don't want it, they're constantly seeing you on the couch, you know. So that time when they are go off [to Young Carers activity] not to worry, because that's what they're there for. So they kind of get used to that, oh mum can be okay when I'm not around.“* (Parent)

Practitioners identified that “selling” the intervention was important and, in doing so, they needed to understand what would be attractive to CYP from an individual perspective:

“*But they would all have their different reasons for coming, won't they? I mean some may come back because they found it was fun, some may come back because they liked the food, some might come back… So everyone's coming from a different angle, young people and even parents. So it's about trying to attract them in some way, and we do that quite well with our activities and stuff, to try and sell it to young people. You think well, I know they like coming because their friend comes, or they like coming because they like that. So they all have different reasons, motivations for coming.”* (Voluntary organization B practitioner)

#### Phase 2: Stakeholder Consensus

As part of our co-development methodology, a stakeholder synthesis day was held. The aim of the synthesis day was to review existing and new research knowledge in order to agree provisional content and delivery preferences for intervention. The synthesis day was open to all individuals currently working with or potentially working with children of parents with severe mental illness in the future. This involved practitioners, academics and managers representing voluntary sector and NHS services attended. During the day, key findings were presented to professional stakeholders alongside existing research. A consensus exercise was used to identify the preferred structure and key components of the finalized intervention, focusing predominantly on implementation and delivery. Tensions between findings and views were explored and acknowledged.

Two qualitative research team members facilitated the day. The aim was to use group consultation to identify agreement and to resolve areas where the evidence was ambiguous or less established. There were three consecutive activities:

1. Listening
2. Identifying gaps and ambiguities
3. Reaching consensus

Nineteen people (9 research team members and 10 non-team individuals) attended the synthesis day. A variety of different professional organizations and roles were represented including those working in academia e.g., health service researchers, psychiatrists, psychologists and a PhD student and voluntary sector organizations e.g., social workers, team managers, business managers and NHS organizations e.g., family therapists, mental health nurses). Attendees were experienced in at least one of the following—working with vulnerable CYP (who may or may not have parents experiencing SMI); working with vulnerable families (where there may or may not be a parent experiencing SMI); working with adults or children with mental health difficulties; or conducting research in the field of adult or CYP mental health.

1. Listening
  Key messages that emerged from existing literature and Phase 1 stakeholder consultation were presented. Attendees were asked to think about what the data implied about what Young SMILES should look like and to make notes that would be useful for activity two.
2. Identifying gaps and ambiguities
  Attendees were asked to reflect on the information from activity one in order to populate a *synthesis matrix*. The matrix aimed to ascertain three areas: intervention format (e.g., content, facilitation, delivery); intervention resources (e.g., training manuals, service resources), and any additional relevant information.
  The two workshop facilitators reviewed the matrix, identifying any contradictory or missing areas. Table 2 presents a summary of aspects that participants were in agreement about and those where some inconsistencies or evidence gaps were acknowledged.
3. Reaching consensus
  In response to the findings from activity two, attendees in four small multidisciplinary groups were asked to discuss one of the four areas where gaps in the evidence or ambiguities had been identified (detailed in Table 2). Participants were told that the aim was to draw upon their own experiences, express their views to the group and listen to those of others to reduce uncertainties and to come to a consensus.
  Summaries of the discussions, including a clear rationale, were fed back verbally to the wider group.
  Following the synthesis day, the planning group consulted with the steering committee and parent representatives prior to finalizing the Young SMILES intervention. The steering committee and parent representatives reviewed and agreed the proposed intervention format, guidance and training materials. Innovation of Theory of Change was used as a guiding methodology for finalizing the change model underpinning the intervention. The intervention was intended to be child-centered and the primary outcome of the intervention, determined a priori at study commissioning stage was enhanced QoL.

**Table 2:** Summary of the outcome of activity two discussions.

**Agreement**	**Inconsistencies/lacking evidence**
**Aim:** Respite, social networking, accessing help, integrated services/wider engagement [Mental Health Education] **Content:** Safety plan, mental health education, signposting, communication skills, practical support for parents **Who:** Discrete age groups **Where:** Transport **Who:** Support worker for parent **Engagement:** Build trust, consolidate peer support, develop between session resources **Resources:** Assessment is crucial, food **Facilitator training:** Group management skills	1. Intervention resources 2. Delivery format • Group composition • How long and over what time period/legacy 3. School/multi-agency liaison and engagement 4. Measures/legislative frameworks • Assessment Progress/success

## Results

**Figure 1:**
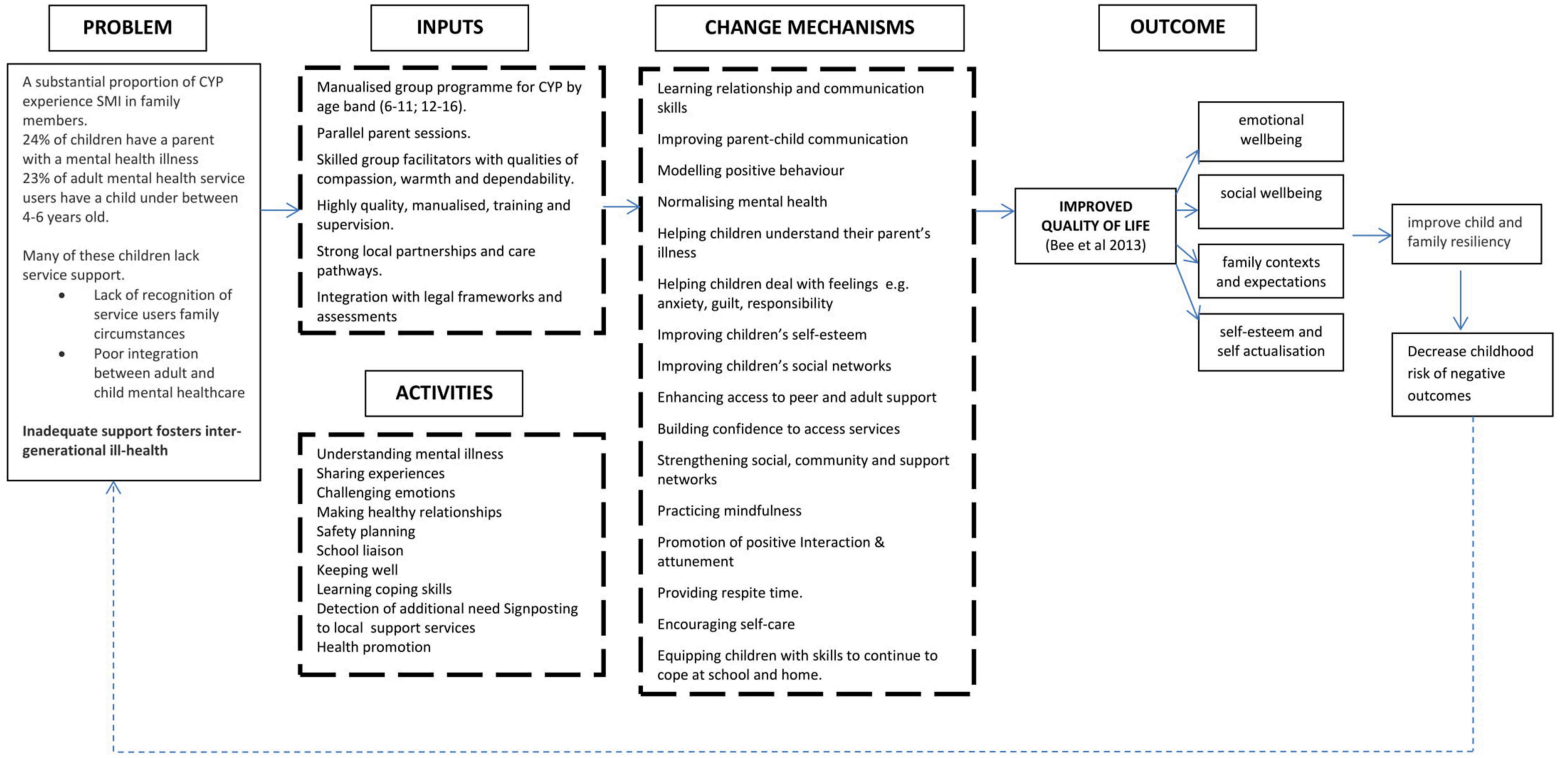
Young SMILES theory of change.

Figure 1 presents the Theory of Change diagram developed as a result of consultation and consensus activities.

Theory of change conceptualizes Young SMILES with respect to the problems faced by CAPRI, Young SMILES inputs and change mechanisms, primary outcome(s) for children and impact upon associated risk of negative outcomes. It additionally takes into account the QoL domains and priorities as determined by CAPRI within previous literature [e.g., (20, 37)]. The change model built on Phase 1 data synthesis and Phase 2 stakeholder consultation and as such was inclusive of multiple perspectives and participants in its design. It required stakeholders to make a distinction between desired and actual outcomes and to identify their desired outcomes before deciding on possible intervention content and processes to achieve those outcomes.

Processes that normalize children's experiences, improve their social and peer support networks and/or enhance their mental health literacy and problem-based coping skills are upheld as important change mechanisms and influenced both the format and content of the Young SMILES intervention. Children's psychological resistance is dependent on these elements to ensure enhanced wellbeing or protection from the impact of potential risk factors by enabling them to gain a better understanding of parental SMI and interact with their family context. Similar priorities have been reported in the literature (39, 40), and empirical work (41). Studies have identified that many CAPRI adopt a caring role for this parent(s), a responsibility that can extend to looking after or supporting other family members (42). The importance of Young SMILES including problem-focused approaches to enhance coping strategies was identified as an important element among stakeholders to empower children to maintain their long-term emotional health. The adoption of a group format, and the inclusion of parent sessions, was hypothesized to strengthen children's and young people's support and encourage child-centered developmental opportunities. Establishing and strengthening social networks is recognized as one potentially effective way of enhancing self-management capacity and well-being (43), and positive family and peer interactions are recognized as important contributors to children's QoL (41, 44).

### Programme Outline

On establishing the theoretical framework, the planning group finalized the intervention outline and facilitators' manual. Resulting data showed some overlap with Family SMILES such as the involvement of parents, the opportunity to meet other children in similar circumstances and increase knowledge, as well as the need for new components. Integration of these two led to Young SMILES being recognized as separate from, but a derivative of, Family SMILES. It was called Young SMILES in recognition of common components.

Young SMILES is a manualised 8-week group programme for CAPRI designed to work with small groups of children/adolescents: it is recommended that a minimum of 4 to a maximum of 6 children/adolescents are involved per group. Its explicit focus is to improve children's health related QoL. The wide age range of Young SMILES groups is split according to the school the young person attends, either primary (6–11) or secondary (12–16). Each group work session is allocated a 2-h time slot, which includes time for a short break and refreshments (with parents/carers) during and after the group. The structure of each session is presented in Table 3.

**Table 3:** Session structure for CYP and parent sessions.

**CPY sessions (week 1–8)**	**Parent sessions (week 4–8)**
• **Welcome:** 10 min •**Warm-up game:** 10 min •**Activity 1:** 10 min •**Made-up family:** 20 min •**Snack break:** 10 min •**Activity 2:** 10 min •**Weekly home activity:** 10 min •**Wind-down game:** 10 min •**Snack and closure:** 30 min	- **Welcome:** 10 min - **Warm-up activity:** 10 min - **Weekly Reflection:** 20 min - **Snack Break:** 10 min - **Feedback from children's sessions:** 20 min - **Wrap-up discussion:** 20 min - **Groups join for end snack:** 30 min

Within each session the following is always covered:

- “Ice-breaker” warm-up activities, including links to previous sessions, to enable the group to recap the main learning points and raise and discuss any issues or questions.
- “Checking in” to identify how things have been since the last session and identifying if any of the CYP need individual time to talk over any particular issues that may have happened since the last session.
- Setting the agenda and objectives for the session—Facilitators set out the session's aims e.g., “today we aim to learn about managing a crisis: who we can contact in a crisis; how to manage our feelings of fear in times of uncertainty; what to do when we think our parent is going into crisis etc.” CYP are given the opportunity to tell the facilitators what they would like to learn or achieve and if they had any anxieties about the session.
- Education and interaction included presentation and discussion of information conveyed via flip charts or drawings, videos, play, creative writing, case studies, and scenarios, all relevant to the learning objectives of the session.
- “Wrapping-up” at the end of the session to elicit feedback on the session, recap on the main learning points, answer questions and agree on activities to be done between sessions and a brief taster of what the next session will cover.
- Getting together as a group for something to eat with in communal space before going home.

At week 4 of the CYP's work group, parallel sessions are offered to the parent/carer who is unwell; and to an additional significant adult in the child's life (identified by the child and their carer) who might attend with the unwell adult, or attend in their place. From week 4 onwards, the CYP and parent groups get together for a snack at the end of the sessions.

**Figure 2:**
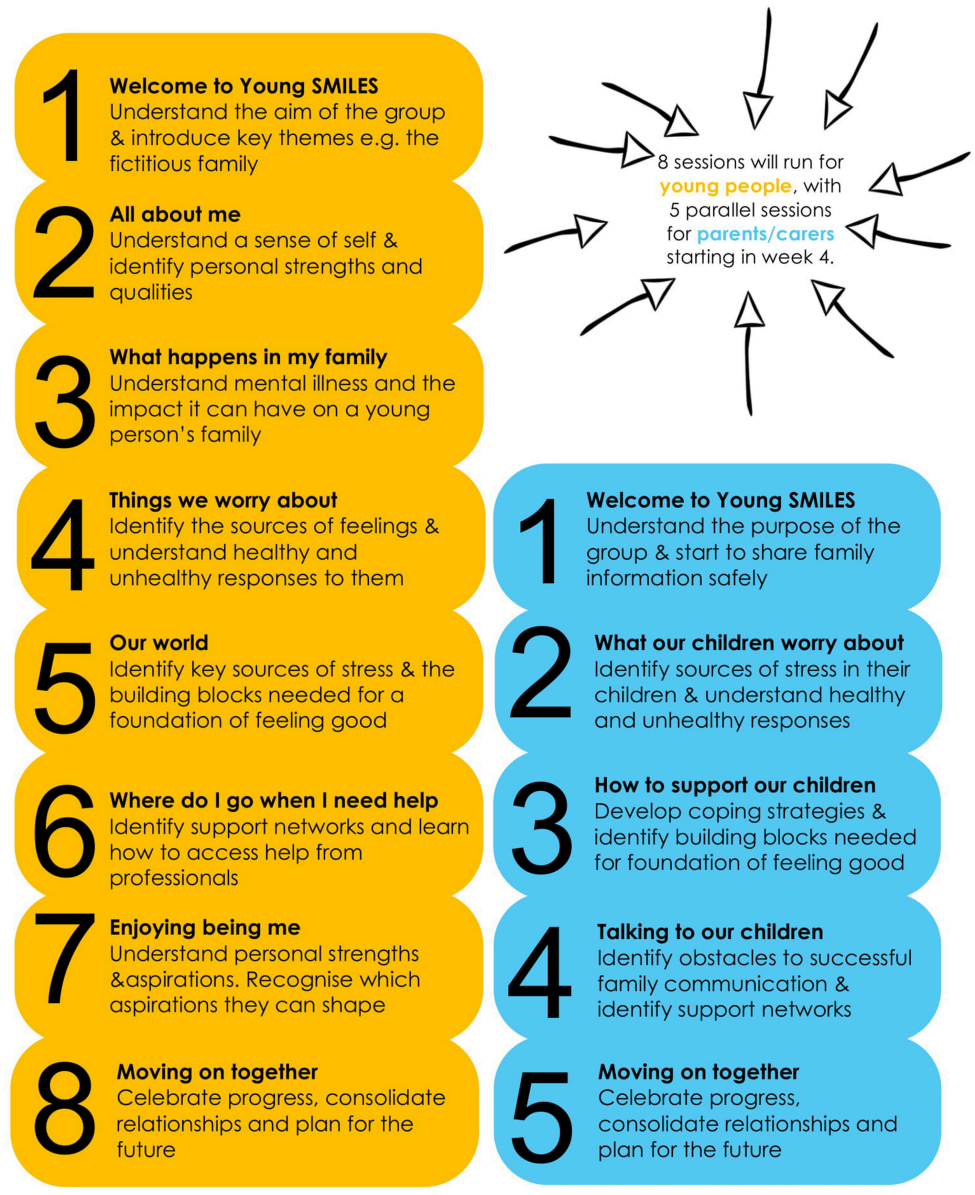
Young SMILES CYP and parent session overview.

Based on stakeholder consultation, we assigned different objectives to each session, but all CYP and parent sessions were underpinned by three emergent themes: mental health literacy, communication and problem-solving skills. Outlined in the facilitator's manual are activities that can be used to achieve the aims of the session. Whilst the overall aims and objectives of each session should not be altered the ways that they can be achieved is flexible to ensure responsiveness to CYP and parent needs. Figure 2 provides an overview of the structure and aims of each proposed CYP (8) and parent (5) sessions. CYP and parent sessions were delivered by two trained facilitators from the NHS or voluntary organizations and held in accessible and acceptable community locations for 2 h each week.

#### Intervention Refinement

The final intervention was presented to service users (parents), practitioners and the trial steering committee for feedback and refinement. Positive feedback was received with individuals indicating that it adhered to the aims of the overall feasibility study and was an accurate reflection of what has been identified via exploration of the research evidence and the views of CYP, parents and professionals. Despite this, some practitioners assigned to delivering the intervention requested that additional step-by-step guidance to conduct the in-session activities be provided to improve their understanding and, in some cases, confidence. A more detailed structure for the in-session activities could also support fidelity to the intervention's objectives and consistency of delivery across sites.

## Discussion

CAPRI are a growing and vulnerable group of multiply deprived young people whose QoL is compromised significantly (2). Little specific provision is available to meet their needs in current services where the focus has been on the parents. In a wholly novel approach, we undertook a series of consultations, focus groups, and a synthesis workshop with CAPRI themselves, with their parents and a broad range of professional stakeholders involved in the support of CAPRI to co-develop a child-centered QoL intervention. Our intervention development aimed to maximize the involvement of all stakeholder groups at the same time as minimizing burden, but further consultation with CYP in the refinement stages of Young SMILES manual could have been beneficial.

There was consistency and overlap between the perceived needs of the CYP, but parents and professional stakeholders did not appreciate their requirements in detail, nor did they appreciate their need for more basic, quotidian support. Although mental health literacy, communication, and problem solving skills emerged as themes for all, CAPRI were clear in wanting peer-focused help and information to understand and manage their parental illness away from their parents in their own ways and in their own space. The young people described feeling isolated socially and in other ways; as well as lacking support and understanding from teachers and schools with a need for greater recognition about their situation by peers, schools and teachers. It was recognized that helping parents understand how and when their illness and behaviors influenced the lives of their children adversely was important but this communication needed some sensitivity. The consistency between perceived needs amongst stakeholders was inconsistent with previous research (19). This could be as the CYP and parents involved were already engaging with services and as a result may have been more informed about the impact that parental mental health was having within the family. The focus of the questions within focus groups and interviews around what an ideal intervention to meet their needs, rather than focusing solely on what their specific needs are, also may have influenced the commonality of responses.

These insights helped us to create a novel, child-centered intervention to deliver away from parents initially in group-based, peer-focused sessions over 8 weeks. It included valued elements for CAPRI such as fun, creative and physical activities and snack times with parents in the latter half of the intervention weeks. Thus, we were able to prioritize CAPRI needs within the wider context of ensuring the feasibility and acceptability of a multidisciplinary team-developed intervention that integrates best practice from mental health and social care.

Young SMILES may provide opportunity for greater collaboration between NHS and voluntary organizations to support CAPRI and mean individuals in different sectors work together and share their knowledge and expertise to meet the needs of this vulnerable and underserved population. Young SMILES, therefore, optimizes the potential value, impact and scalability across statutory services none of whom traditionally are not targeting this group which occupies a space between the health and social care interface.

Our future work lies in piloting the broader acceptability and feasibility of the new programme and testing our ability to deliver it to scale within a randomized controlled trial method for future evaluation of clinical and cost effectiveness.

## Conclusions

There is a clear need for a child-centered, specific and focused approach to supporting vulnerable young people living with severe parental mental illness across the UK and globally (2). We have recognized the need to consult closely with children themselves and have co-developed an intervention shaped by their input and understanding with an emphasis on peer support separate to the support their parents receive. Future formal evaluation of Young SMILES will help to consolidate these close links with CAPRI keeping them at the center of solutions to the difficulties they face on a daily basis.

## Ethics Statement

The study was approved by North West - Greater Manchester East Research Ethics Committee (Ref: 16/NW/0207 13th April 2016). Approval was also obtained from Barnardo's Research Ethics Committee (BREC) 26th July 2016. All participants gave written informed consent in accordance with The Declaration of Helsinki.

## Author Contributions

JG, PB, DH, LG, CC, and KA participated in the development of Young SMILES. JG, PB, and AK analyzed the qualitative data. JG, PB, KA, LG, and HH prepared the manuscript. All the authors read and approved the final manuscript.

### Conflict of Interest Statement

The authors declare that the research was conducted in the absence of any commercial or financial relationships that could be construed as a potential conflict of interest.
